# Supplementary material for: A Novel, Tumor-Induced Osteoclastogenesis Pathway Insensitive to Denosumab but Interfered by Cannabidiol
Source: Int J Mol Sci. 2019 Dec 9;20(24):6211. doi: 10.3390/ijms20246211 (PMC6940789; doi:10.3390/ijms20246211)
Supplement: Supplementary file 1 [file ijms-20-06211-s001.pdf]

A Novel Tumor Induced Osteoclastogenesis Pathway Insensitive to Denosumab but Interfered by Cannabidiol

Maiko Tsuchiya [1], Kou Kayamori [1], Akane Wada [2], Motohiro Komaki [3], Yae Ohata [2#], Miwako Hamagaki [1], Kei Sakamoto [1] and Tohru Ikeda [1*]

[1] Department of Oral Pathology, Graduate School of Medical and Dental Sciences, Tokyo Medical and Dental University, 1-5-45, Yushima, Bunkyo-ku, Tokyo 113-8549, Japan
[2] Department of Oral Diagnostic Pathology, Graduate School of Medical and Dental Sciences, Tokyo Medical and Dental University, 1-5-45, Yushima, Bunkyo-ku, Tokyo 113-8549, Japan
[3] Department of Highly Advanced Stomatology (Periodontology), Graduate School of Dentistry, Kanagawa Dental University, 3-31-6 Tsuruya-cho, Kanagawa-ku, Yokosuka-city, Kanagawa 221-0835, Japan
* Correspondence: tohrupth.mpa@tmd.ac.jp Tel.: +81-3-5803-5451
# Present address: Department of Medical Biochemistry and Microbiology, Uppsala University, Box 582, SE-75123 Uppsala, Sweden

Supplementary Information

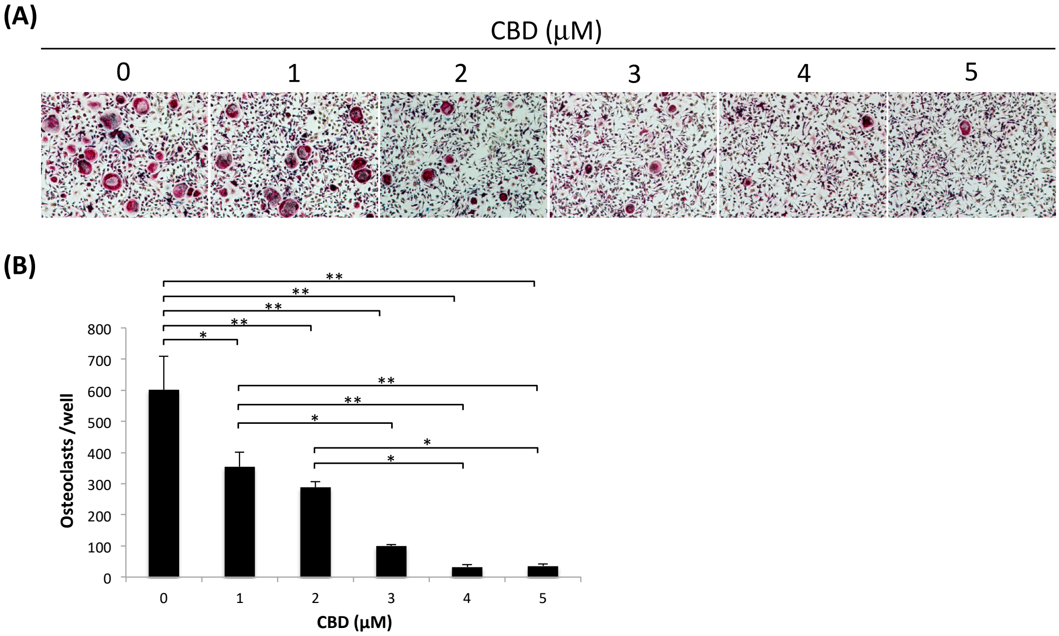

Figure S1. Optimization of CBD concentrations to inhibit osteoclastogenesis induced by the coculture. (A) Representative views of TRAP staining 4 days after the application of each concentration of CBD to a coculture of OPCs and 3A cells. (B) Quantitative data on the number of induced osteoclasts. Values are the mean ± SEM of 3 wells. Values are the mean ± SEM of 3 wells. $^{**}P < 0.001$, $^{*}P < 0.05$ (one-way ANOVA with the Tukey-Kramer method).

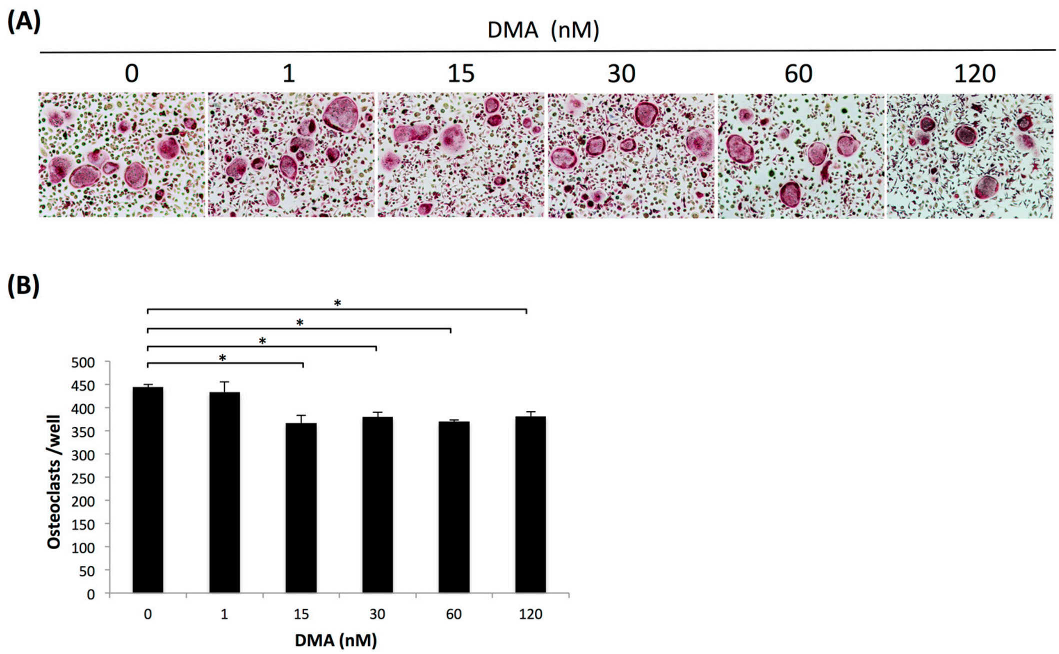

Figure S2. Optimization of DMA concentrations to inhibit osteoclastogenesis induced by the coculture. (A) Representative views of TRAP staining 4 days after the application of each concentration of DMA to a coculture of OPCs and 3A cells. (B) Quantitative data on the number of induced osteoclasts. Values are the mean ± SEM of 3 wells. *$P < 0.05$ significantly different from that without the application of DMA (one-way ANOVA with the Tukey-Kramer method).

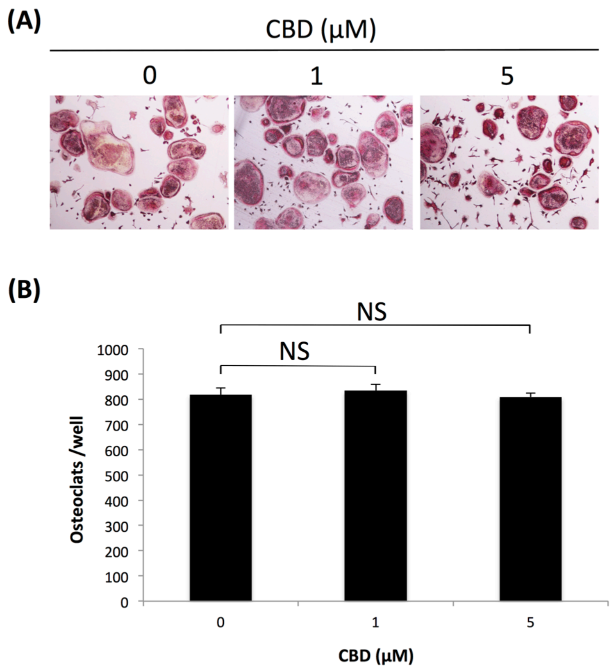

Figure S3. Effects of CBD on osteoclastogenesis induced from OPCs by RANKL. (A) Representative views of TRAP staining 4 days after the application of each concentration of CBD to OPCs further treated with

100 ng/mL of RANKL. (B) Quantitative data on the number of induced osteoclasts. Values are the mean ± SEM of 3 wells. NS: not significant (one-way ANOVA with the Tukey-Kramer method)

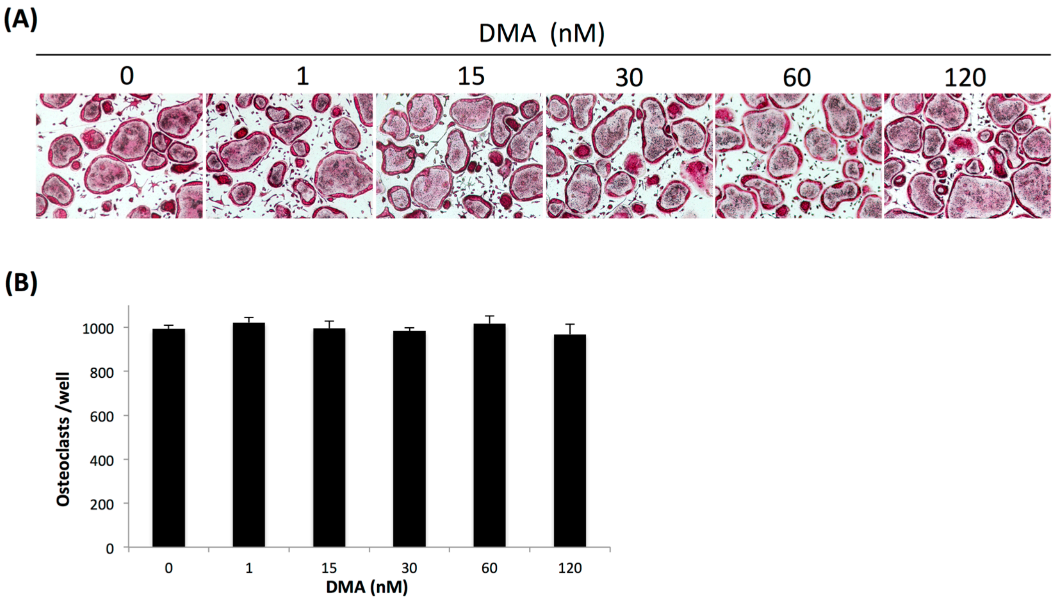

Figure S4. Effects of DMA on osteoclastogenesis induced from OPCs by RANKL. (A) Representative views of TRAP staining 4 days after the application of each concentration of DMA to OPCs further treated with 100 ng/mL of RANKL. (B) Quantitative data on the number of induced osteoclasts. Values are the mean ± SEM of 3 wells. No significant difference was observed in the number of osteoclasts (one-way ANOVA with the Tukey-Kramer method).
